# Validity and reliability of a Chinese version of the Bergen Work Addiction Scale

**DOI:** 10.3389/fpsyg.2023.1029846

**Published:** 2023-05-02

**Authors:** Yuanyuan Liu, Hongjun Tian, Xinying Chen, Feng Jia, Ranli Li, Yun Sun, Langlang Chen, Jingjing Zhu, Deguo Jiang, Chuanjun Zhuo

**Affiliations:** ^1^Department of Psychosomatic Medicine, Tianjin Chest Hospital, Tianjin, China; ^2^Department of Psychiatry, Tianjin Fourth Center Hospital, Tianjin, China; ^3^Department of Psychiatry, Tianjin Anding Hospital, Tianjin, China; ^4^Department of Psychiatry, Wenzhou Seventh People’s Hospital, Wenzhou, China

**Keywords:** work addiction, Bergen Work Addiction Scale, Chinese, reliability, validity

## Abstract

**Background:**

Work addiction (WA), which can impair personal relationships, engagement in recreational activities, and/or health, is a behavioral addiction. A tool for the early detection of WA in China is needed.

**Objective:**

The aim of this study was to develop and determine the validity and reliability of a Chinese version of the Bergen Work Addiction Scale (C-BWAS).

**Methods:**

Two hundred social workers who provided post-discharge services for adolescents with non-suicidal self-injury (NSSI) were enrolled in this study. The construct validity of the C-BWAS was assessed by confirmatory factor analysis (CFA). Criterion validity was assessed by conducting Pearson correlation analyses of C-CWAS scores with Hamilton Depression Scale (HAM-D) and Hamilton Anxiety Scale (HAM-A) scores. Cronbach’s α and the intra-class correlation coefficient (ICC) were used to evaluate the reliability of the C-BWAS.

**Results:**

CFA confirmed a one-dimensional structure of the C-BWAS with good construct validity indices [comparative fit index (CFI) = 0.964, Tucker–Lewis index (TLI) = 0.951, root-mean-square error of approximation (RMSEA) = 0.079, and minimum discrepancy Ĉ/degrees of freedom (Cmin/DF) = 0.362]. The standardized regression weights ranged from 0.523 to 0.753. All C-BWAS items loaded on one major factor (loading weights, 0.646–0.943). Coefficients of correlation between C-BWAS scores and HAM-D and HAM-A scores were 0.889 and 0.933, respectively. The Cronbach’s α coefficient and ICC for the instrument was 0.837 and 0.905, respectively.

**Conclusion:**

The presently developed C-BWAS showed very good reliability and acceptably validity. It can be employed as a useful tool for assessing WA severity in social workers who provide post-discharge services for adolescents with NSSI.

## Introduction

Excessive commitment to one’s job is characterized as the confusion of obsessive thoughts and fears for dedication to one’s work and personal discipline (Park, 2021). Social workers who provide post-discharge services for adolescents with non-suicidal self-injury (NSSI) may be incentivized to show initiative and have proactive attitudes, but they can become overly engaged in their jobs (Ekstedt et al., 2022). Most of these workers have high levels of empathy, and adolescents with NSSI tend to require their services frequently, as many of these adolescents feel helpless and lonely. These reciprocal factors converge to increase the risk of work addiction (WA) for these social workers.

WA is distinctively characterized by the following symptoms: obsessive thinking about work and success; intense fear of failing at one’s job; persistent anxiety and rumination about one’s job performance; defensive thoughts or reactions to others’ concerns or questions about one’s work; becoming overwhelmed with negative feelings; and avoidance of confronting other challenges in one’s life, such as grief or trauma (Quinones and Griffiths, 2015). Although people with WA can often hide the above symptoms from others, externally recognizable signs of WA include volunteering to work extra hours unnecessarily (especially without overtime pay), loss of sleep (especially reallocation of sleep time to work), cutting back on non-work activities that one has historically enjoyed to work more hours, and social isolation. An assessment tool to discriminate WA from normal work engagement is needed to protect workers’ physical and mental health.

Work engagement, operationally defined as a positive work-related state of fulfillment characterized by vigor, dedication, and absorption or concentration at work, can be assessed with the Utrecht Work Engagement Scale (UWES-9) (Schaufeli et al., 2002; Leiter and Bakker, 2010; Andreassen et al., 2012; Domínguez-Salas et al., 2022; González-Rico et al., 2022; Odagami et al., 2022). The concept of work engagement has been further described as a persistent affective-cognitive state that is positively associated with vigor, or a high level of energy, as well as with will and mental resistance; dedication, or feeling enthusiastic about and challenged at work (Crowe et al., 2022). Meanwhile, the term absorption describes a state of concentrating fully and exclusively on tasks being performed (Crowe et al., 2022).

Unlike work engagement, which is a positive factor that facilitates achievement and the attainment of positive emotional states such as happiness (Smith et al., 2021), WA a stable tendency to engage in work excessively and compulsively (Andreassen, 2014), to the point that one experiences a loss of control over working activity boundaries that persists over time (Atroszko et al., 2017; Atroszko and Grifths, 2017; Griffiths et al., 2018). Moreover, WA tends to lead to personal distress, strained relationships, and impaired functioning due to stress, anxiety, and/or depressive symptoms (Quinones and Griffiths, 2015). WA has been assessed with the Dutch Work Addiction Scale (DUWAS) and Bergen Work Addiction Scale (BWAS). DUWAS and BWAS scores can provide useful information for planning interventions aimed at alleviating the mental anguish associated with WA, with the DUWAS providing information regarding behavioral features and the BWAS providing information related to mental suffering.

The DUWAS is a 10-item scale with two 5-item subscales: Working Excessively (WE) and Working Compulsively (WC). Each DUWAS item is rated on a 1 (almost never) to 4 (almost always) scale. The scale results are considered to be indicative of WA when a respondent has a total DUWAS score (WE + WC sub-scores) in the upper quartile together with mean WC-item scores and mean WE-item scores that exceed 2.2 and 2.8, respectively (Del Líbano et al., 2010).

The BWAS, developed in 2012 by Andreassen et al. (2012), is a cost-free 7-item psychometric tool used to probe WA symptoms in the past 12 months. An item is included for each of the core addiction components (salience, mood modification, tolerance, withdrawal, conflict, relapse, and problems) and each item is responded to on a 1 (never) to 5 (always) Likert scale. Scores of 4 (often) or 5 (always) on four or more items is regarded as suggesting that the respondent is a workaholic. Andreassen and colleagues found that the BWAS has a unidimensional structure with all items loading in one factor, work addiction. BWAS also has previously been validated in other countries, such as Italy, and the BWAS has been used successfully in prior studies to measure work addiction (Molino et al., 2002; Andreassen et al., 2016; Atroszko et al., 2016; Lichtenstein et al., 2019; Fekih-Romdhane et al., 2022). The original English-language BWAS has been used to help workers make adjustments to protect their health. The aims of the present work were firstly to develop a Chinese version of the Bergen Work Addiction Scale (C-BWAS) and secondly to assess its validity and reliability with a sample of Chinese social workers employed at a mental health center who provide post-discharge services for adolescents with NSSI (Table 1).

**Table 1:** C-BWAS item loadings on a single factor.

Item	Loading	95% CI
1. Thought of how you could free up more time to work?	0.752	0.652–0.588
2. Spent much more time working than initially intended?	0.885	0.700–0.922
3. Worked in order to reduce feelings of guilt, anxiety, helplessness 4. and depression?	0.943	0.842–1.00
5. Been told by others to cut down on work without listening to them?	0.646	0.555–0.789
6. Become stressed if you have been prohibited from working?	0.841	0.748–0.980
7. Deprioritized hobbies, leisure activities, and exercise because of your work?	0.658	0.490–0.852
8. Worked so much that it has negatively influenced your health?	0.888	0.603–0.956

Respondents were instructed to respond to each item on the following rating scale: 1 = never, 2 = rarely, 3 = sometimes, 4 = often, 5 = always. C-BWAS, Chinese version of the Bergen Work Addiction Scale; CI, confidence interval.

## Materials and methods

### Participants

Two hundred social workers (45 men, 155 women) who provided post-discharge services for adolescent patients with suicidal ideation in Wenzhou Seventh People’s Hospital, Tianjin Fourth Center Hospital, and Tianjin Chest Hospital, China were recruited and enrolled in this study by convenience sampling (response rate, 100%). The mean age of the participants was 29.5 ± 3.0 years (range, 24–44 years). The mean duration of experience that the participants had in this role was 4.5 ± 1.2 years (range, 3–7 years). The ethics committee of Tianjin Fourth Center Hospital approved the present study (no. 20190105-JWK), and all participants provided written inform consent.

Clinically significant anxiety and depression in participants were diagnosed by a senior psychiatrist employing the 17-item Hamilton Depression Scale (HAM-D) and the Hamilton Anxiety Scale (HAM-A) (Hamilton, 1959, 1960) in accordance with the Diagnostic and Statistical Manual of Mental Disorders (edition IV). Briefly, a HAM-A score ≥ 14 and a HAM-D score ≥ 17 were considered indicative of the presence of clinically significant anxiety symptoms and clinically significantly depression symptoms, respectively.

### Scale translation

Six native Chinese-speaking senior psychiatrists with English fluency from the psychiatry departments of Tianjin Fourth Center Hospital and Wenzhou Seventh People’s Hospital forward-translated the original BWAS into Chinese. A native English-speaking psychiatrist with Mandarin Chinese fluency (Dr. S. Patricia Chou, Chief of NIAAA) then back-translated the draft C-BWAS. All seven translators then met and compared the original BWAS and the back-translated version to detect and address any inconsistencies. For any discrepancy that emerged, clarification was sought directly from the original BWAS developers to ensure the maintenance of conceptual validity.

### C-BWAS assessment

The instrument’s criterion validity was evaluated by way of Pearson correlation analyses with the 17-item HAM-D and the HAM-A (Hamilton, 1959, 1960). The construct validity of the unidimensional structure was assessed by confirmatory factor analysis (CFA). C-BWAS’ internal consistency was assessed by calculating the Cronbach α coefficient. One-month test–retest reliability was assessed using the intra-class correlation coefficient (ICC) (Heidari and Feizi, 2018; Liu et al., 2020; Dang et al., 2021; Yuliana et al., 2022).

### Statistical analysis

Descriptive statistics (i.e., *t*-test, internal consistency coefficients, and convergent validity) and normality testing were conducted in SPSS, version 23.0 (IBM, United States).

To verify the structural model of the BWAS, CFA was conducted with a weighted least squares, mean, and variance adjusted estimator that enables treatment of ordinal data in Mplus, version 7.4 (Schreiber, 2008; Andreassen et al., 2011; Hair et al., 2019; Solikhah et al., 2023). In the CFA, model fitness was determined based on the comparative fit index (CFI), Tucker–Lewis index (TLI), and root-mean-square error of approximation (RMSEA) value; all three of these indices are well established as effective and reliable indicators (Schreiber, 2008; Andreassen et al., 2011; Hair et al., 2019; Solikhah et al., 2023). The criteria for an acceptable model fit were: CFI > 0.90, TLI > 0.90, and RMSEA <0.08 (Schreiber, 2008; Andreassen et al., 2011; Hair et al., 2019; Solikhah et al., 2023). As an additional measure of model fitness, we calculated the quotient of the minimum discrepancy, Ĉ, and degrees of freedom (DF), written Cmin/DF. A |Cmin/DF value| < 3 indicated an acceptable fit.

To investigate a hypothesized BWAS structure, a theoretical 1-factor (mental suffering) structure was imposed while treating age and gender as covariates. CFA was used to assess the construct validity of C-BWAS. HAM-D and HAM-A were used as references for convergent validity testing, such that significant correlation coefficients for BWAS versus HAM-D outcomes and BWAS versus HAM-A outcomes were considered supportive of convergent validity.

The C-BWAS’ internal consistency was assessed by calculating the Cronbach α coefficient. One-month test–retest reliability was assessed by calculating the ICC.

## Results

### Mental suffering states of the participants

The study sample of 200 social workers included 105 (52.5%; 33 men and 72 women) who were found to have anxiety symptoms requiring medical intervention (HAM-A score ≥ 14) and 62 (31.0%; 25 men and 37 women) who were found to have depression symptoms requiring medical intervention (HAM-D score ≥ 17).

### C-BWAS properties

#### Construct validity

CFA demonstrated that the C-BWAS has an adequate goodness of fit based on predetermined fit criteria. The values obtained for the C-BWAS for those predetermined fit parameters were: χ^2^/df = 2189.422, df = 258; CFI = 0.964, TLI = 0.951, AGFI = 0.947, RMSEA = 0.079 (90% CI, 0.073–0.088), SRMR = 0.078. |Cmin/DF value| =1.0252. The adequacy index of sampling was 0.799 and the Bartlett’s sphericity test result was statistically significant (χ^2^ = 5266.452; df = 217; *p* < 0.001). All items in the model were loaded substantially on their respective factors, except for those factor-constraint items that could not be tested for significance; all item-domain correlation coefficient values were 0.40. This datum indicates that C-BWAS has good construct validity.

#### Criterion validity

C-BWAS scores correlated robustly with HAM-D (*r* = 0.889, *p* < 0.01) and HAM-A (*r* = 0.933, *p* < 0.01) scores. These data indicate that the C-BWAS has good criteria validity.

### Reliability

Regarding internal consistency, the total Cronbach’s α coefficient for the C-BWAS was 0.837. For 1-month test–retest reliability, the ICC value was 0.905.

## Discussion

The C-BWAS developed in this study was found to have acceptable psychometric properties and was shown to be a unidimensional scale, a finding that is consistent with the theoretical structure of the original English-language version of the BWAS. The present data thus indicate that the C-CBWAS is an appropriate tool for early screening of social workers for WA.

The high degrees of correlation observed for C-BWAS scores with both HAM-D and HAM-A scores suggest that individuals with WA are prone to depression and anxiety. Indeed, large proportions of the social workers participating in this study had these conditions. The prevalence of depression and anxiety in this convenience sample was greater than in the general population (Hook et al., 2012; Lu et al., 2021).

Work is necessary and is affirming for many people, but workers should create healthy work habits and attitudes to avoid WA development, which can damage physical and mental health. For example, hyper-engagement in work could cause social function disturbance and even physical illness. Chronic stress as a consequence of WA may lead to other disorders, including depression, anxiety, and substance use disorders, such as nicotine dependence and alcohol or sedative abuse (American Psychiatric Association, 2013; Hauk and Chodkiewicz, 2013; Durand-Moreau et al., 2018; Kang, 2020; Kun et al., 2020; Cossin et al., 2021; Li et al., 2021; Eason et al., 2022; Fekih-Romdhane et al., 2022; Zeng and Liu, 2022). Hence, screening for WA can help to protect social workers’ physical and mental health. The C-BWAS is a useful tool for such screening.

WA is a public health concern that can lead to negative health outcomes (Andreassen et al., 2014). However, the progression from WA mental health concerns have not been clarified (Andreassen et al., 2013). Estimates of WA prevalence in the literature are in the range of 8.3 ~ 10.0% (Dutheil et al., 2020; Li et al., 2020). A multitude of negative effects of WA have been acknowledged in the literature, including elevated job stress, impaired job performance, depression, sleep disorders, and increased work–family conflicts (Schaufeli et al., 2008; Andreassen, 2014; Quinones and Griffiths, 2015; Andreassen et al., 2017). Indeed, initiatives aimed at WA prevention would be timely given that WA is increasingly being recognized as a significant contributor to pathologies that constitute major components of the global burden of disease (Schaufeli et al., 2008; Andreassen et al., 2013, 2017; Andreassen, 2014; Quinones and Griffiths, 2015; Dutheil et al., 2020; Li et al., 2020). To protect their workers, employers should be aware of WA risk factors, such as high job demands (Schaufeli et al., 2008; Andreassen et al., 2013, 2017; Andreassen, 2014; Quinones and Griffiths, 2015; Dutheil et al., 2020; Li et al., 2020). Other preventive strategies, such as social support programs, may benefit workers at risk of WA. In the context of WA prevention, employers (or their human resources departments) of Chinese speaking workers, in China as well as in Malaysia, Indonesia and Singapore, may use the C-BWAS to determine the prevalence of WA in their units and to identify affected and at-risk workers who should be targeted for intervention. Furthermore, on the national level, governments of Chinese language speaking populations can use the C-BWAS as a tool to monitor domestic WA prevalence patterns and thereby enact preventative and early intervention strategies.

This study had several limitations. First, our ability to assess the convergent validity of the C-BWAS was limited by the lack of another similar instrument in Chinese. We did correlate our C-BWAS results to our HAM-D/A results, but those correlations need to be supplemented in future research with further convergent validity analyses, particularly among general population workers, including workers with neuropsychiatric diagnoses. Second, we obtained correlation coefficient values for correlations of C-BAWS scores with HAM-D/A scores that were very high, exceeding coefficients obtained for correlations of the original BAWS with another WA scale. These strong correlations do however support the supposition that social workers, who are regularly exposed to negative mood interactions, experience serious mental health symptoms. Third, it should be noted that the participants enrolled this study were recruited from a select occupational group with features that may not generalize to workers in other occupations. Finally, because we did not make clinical diagnoses of WA, we could not ascertain a C-BAWS cut-off score for WA screening. Further work in China is warranted to fill this knowledge gap.

## Conclusion

The C-BWAS developed in this study showed good validity and reliability for the assessment of WA severity among Chinese social workers providing post-discharge services for adolescents with NSSI. The C-BWAS can be used for the early detection of WA, which may prevent the development of comorbidities and burnout among workers in China and other Chinese speaking countries.

## Data availability statement

The original contributions presented in the study are included in the article/supplementary material, further inquiries can be directed to the corresponding authors.

## Ethics statement

The studies involving human participants were reviewed and approved by the Tianjin Fourth Hospital. The patients/participants provided their written informed consent to participate in this study. Written informed consent was not obtained from the individual(s) for the publication of any potentially identifiable images or data included in this article.

## Author contributions

YL, RL, YS, LC, and JZ conceived and designed the research. CZ, HT, and DJ collected the data and conducted the research. YL and DJ analyzed and interpreted the data. RL, YS, LC, and JZ wrote the initial draft of the manuscript. CZ and HT revised the manuscript. DJ and HT had primary responsibility for the manuscript’s final content. All authors read and approved the final version of the manuscript.

## Funding

This work was sponsored by grants from the National Natural Science Foundation of China (82171503 and 81871052 to CZ), the Key Projects of the Natural Science Foundation of Tianjin, China (17JCZDJC35700 to CZ), the Tianjin Health Bureau Foundation (2014KR02 to CZ), and the Tianjin Science and Technology Bureau (15JCYBJC50800 to HT).

## Conflict of interest

The authors declare that the research was conducted in the absence of any commercial or financial relationships that could be construed as a potential conflict of interest.

## Publisher’s note

All claims expressed in this article are solely those of the authors and do not necessarily represent those of their affiliated organizations, or those of the publisher, the editors and the reviewers. Any product that may be evaluated in this article, or claim that may be made by its manufacturer, is not guaranteed or endorsed by the publisher.
